# Ultrasound measurement of perirenal adipose tissue indicates cardiovascular disease, but standardisation is needed: A systematic review

**DOI:** 10.1002/ajum.12407

**Published:** 2024-10-20

**Authors:** Victoria J. A. Baumann, Richard Banati, Jillian L. Clarke

**Affiliations:** ^1^ Discipline of Medical Imaging Science, Faculty of Medicine and Health The University of Sydney Camperdown New South Wales Australia

**Keywords:** adipose tissue, cardiovascular disease, ectopic fat, pararenal fat, perirenal fat, subcutaneous fat, ultrasound, visceral fat

## Abstract

**Introduction:**

In both highly industrialised and developing countries, obesity is reaching epidemic proportions and increasingly becoming a critical indicator of general morbidity, cardiovascular disease (CVD) and renal dysfunction. A promising trend in detection and management of obesity is the measurement of perirenal adipose tissue (PRAT), increasingly recognised as a metabolically active endocrine organ in itself. Its measurement by ultrasound is used increasingly to indicate visceral obesity and its clinical management. This review synthesises current techniques for measuring PRAT and its potential use as an indicator of CVD.

**Methods:**

We included clinical studies published between 2010 and 2023, investigating the current practice and use of specific ultrasonographic techniques and assessed the reliability and accuracy of included papers. The risk of bias was assessed using the Downs and Black Checklist, and the methodological quality examined using the Grade of Recommendations, Assessments, Development and Evaluation.

**Results:**

It found, PRAT measures are predictive of CVD risk factors and the accuracy of ultrasound is comparable to CT and MRI, but there is no consistency in ultrasound technique. The lack of any randomised control trials and the use of 20 different non‐standardised ultrasound techniques across the 21 studies resulted in inconsistent and imprecise clinical observations and interpretations, which decreased the overall quality of the studies.

**Conclusion:**

This review found the inclusion of ultrasound measures in routine abdominal imaging potentially invaluable but demonstrates the need for standardisation of the perirenal fat ultrasound measuring technique to improve reproducibility and reliability.

## Introduction

This systematic review investigates the current literature into the ultrasound measurement of perirenal adipose tissue (PRAT) as an indicator of cardiovascular disease (CVD). Its aim was to understand current practices, techniques involved and its reliability and accuracy compared with other imaging modalities.

### Traditional indices of obesity

The clinical assessment of obesity has traditionally relied on the measurement of waist circumference and the calculation of the body mass index (BMI); however, these measurements are increasingly seen as an inadequate assessment of health risk.^1^ They do not differentiate between subcutaneous, visceral or ectopic fat, or indeed fat and non‐fat tissues, such as muscle, which are all components of whole‐body mass.^2^

### PRAT an endocrine organ

Perirenal adipose tissue, a type of visceral fat, may have a closer association with cardiovascular and renal disease than other visceral deposits in obesity. It is a metabolically active and complex endocrine organ with both dormant and active adipocytes and pre‐adipocytes, sympathetic nerve endings, vascular structures and inflammatory cells of different types. It has been shown to be associated with chronic renal disease (CRD) and cardiovascular disease,^3^ hypertension^4^ and atherosclerosis.^5^

**Figure 1:**
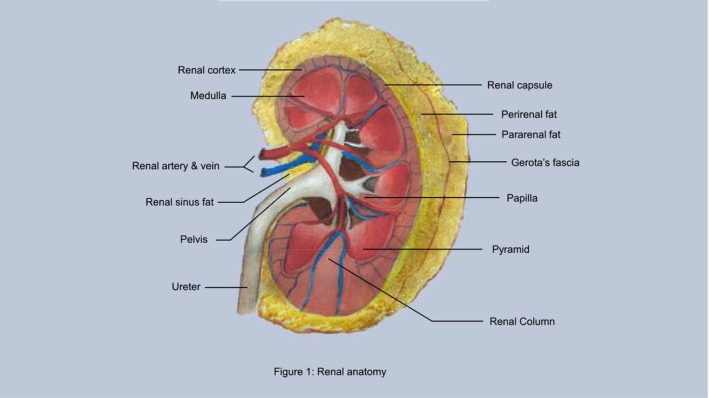
Renal adipose tissue anatomy.^8^

The kidneys are retroperitoneal organs, enveloped by connective tissue fascia, encasing renal fat and the kidneys themselves. Perirenal adipose tissue is located between the renal capsule (adherent to the kidneys) and the renal or Gerota's fascia. Contiguous with this is renal sinus fat, a perivascular fat confined to the renal sinus and the renal fibrous membrane.^6^ Key to the study of excessive PRAT is the understanding that Gerota's fascia is a tough, tight, fibrous membrane with limited ability for expansion. In close proximity to the renal vessels, firmly encapsulated PRAT has been hypothesised to have a mechanical compressive effect on the renal parenchyma. Posteriorly lies the white, inactive fat of the pararenal body, known as pararenal fat. Renal sinus fat, too, has been shown to be associated with increased vascular resistance, possibly by a similar mechanical compression, which may cause hypertension and CRD development, though the exact mechanism is not fully understood.^7^ These fat deposits are shown in Figure 1.^8^

Prolonged hypertension is the ‘predominant risk factor’ in the development of CVD^9^ which includes atherosclerotic and valvular disease, heart failure, atrial fibrillation, cerebrovascular and chronic kidney disease, retinal and metabolic disease.

Lamb^10^ discusses non‐invasive quantification of visceral and ectopic fat compartments, using ultrasound, CT and MRI, but note the latter two ‘are more expensive and less accessible than ultrasonography’ (p. 136).

As an endocrine organ, PRAT is associated with hypertension, obesity, chronic renal disease and tumour progression.^11^ Obesity causes the overproduction of free fatty acids, exacerbating chronic inflammation by systemic or local secretion of cytokines. These promote endothelial dysfunction, known to be an initiator of atherosclerosis and CVD. Katsiki *et al*.^12^ concluded that excessive perirenal adiposity increases the risk of coronary heart disease and hypertension through adipokine secretion, fat–kidney interactions and a neural reflex.

### Imaging

An increase in PRAT thickness, most accurately measured in vivo by radiology,^11^ is associated with high cardiovascular and/or metabolic risk.^4^, ^13^ Non‐invasive modalities such as ultrasound, CT and MRI all have practical implications, but not all are available in all settings. CT and MRI are the gold standards but require equipment that is not easily transported nor suited for large‐scale use. Ultrasound, however, is comparatively more affordable and portable, is well‐tolerated by patients and involves no ionising radiation. In regional and remote communities, ultrasound may be the initial, or indeed only, available imaging choice. It is an attractive option for clinical use in low‐ and middle‐income countries (LMICs) for both in‐patient and out‐patient use. The number of LMICs that reported ultrasound use has increased 24% since 2010 from 50 to 62 countries.^14^

In Australia, high numbers of abdominal ultrasound examinations are performed annually. The addition of one measurement of the PRAT thickness, within routine imaging, would add no perceptible increase in examination time, while a finding of excessive fat could well‐initiate life‐saving interventions for a patient at risk. With minimal additional training, ultrasound could be used in a timely and cost‐effective manner, with widespread delivery.

## Methods

### Database search

After registering with PROSPERO^15^, the Preferred Reporting Items for Systematic Reviews and Meta‐Analyses protocol (PRISMA),^16^ was followed. The search was then run, and subsequently updated on 31 October 2023. Data bases searched were PubMed, Medline, Ovid, Scopus, Web of Science, Cinahl and Google Scholar.

Keywords included adipose tissue, CVD, diagnostic imaging, ectopic fat, PRAT and perirenal fat. The full list of search terms is included in Appendix S1. Articles were uploaded onto Endnote X9 reference manager and imported into the Covidence Software package^17^ to assist with removal of duplicates, screening of references and data extraction.

### Study selection and data extraction

All study types were eligible for inclusion. Studies excluded were those not published in the English language, animal or post‐mortem studies, paediatric populations or studies where a full text was not available. Conference proceedings and grey literature were excluded.

**Figure 2:**
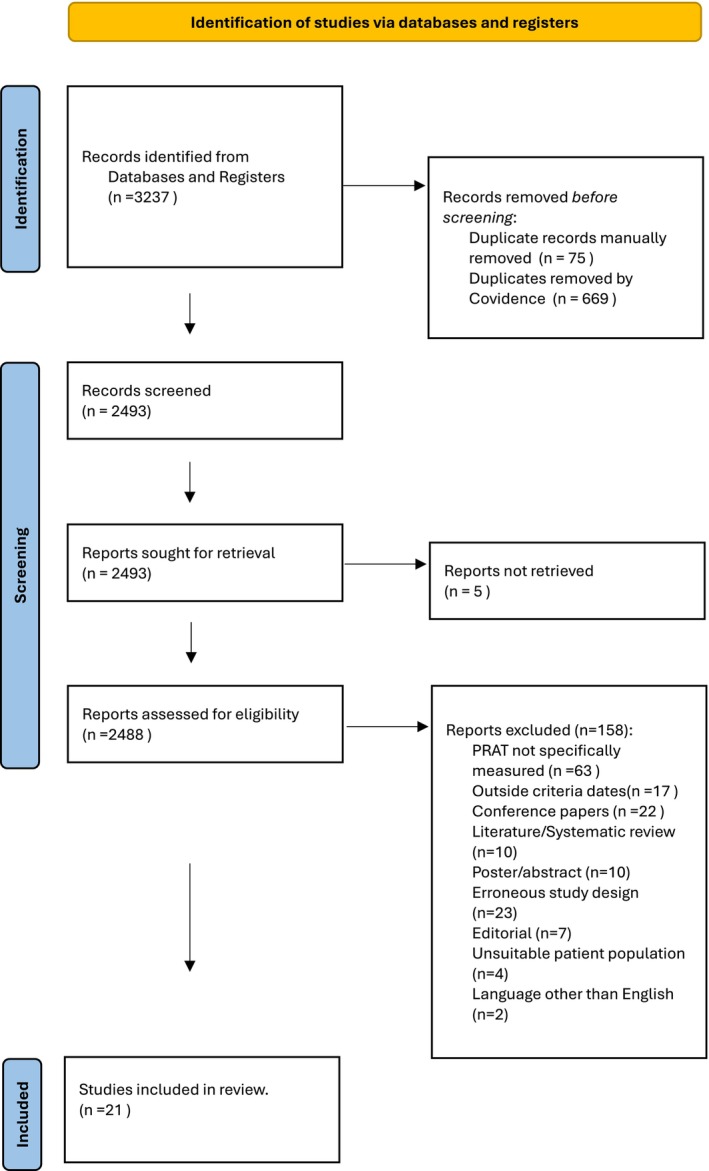
PRISMA flow diagram.

The searches yielded 3237 studies. Articles were reviewed independently by two researchers, VB and JC. Titles and abstracts were screened for eligibility followed by full‐text review, resulting in the inclusion of 21 studies: 6 cohort, 5 case control and 10 cross section studies. The total number of participants across all included studies was 2053. The PRISMA^15^ flow diagram showing identification of studies via databases and registers is shown in Figure 2.

A full list of excluded studies is found in Appendix S2 (Table 1).

**Table 1:** Summary of characteristics of included studies (n = 21).

Author	Year	Country	Study design	Number of subjects	Ultrasound measurement technique for perirenal fat layer thickness	Main findings	Ultrasound operator	Link postulated between PRAT thickness and CVD	Images provided
Alempijevic *et al*.	2011	Serbia	Case‐control	90	Posterior right renal wall in the posterior perinephric space	Ultrasonography is ‘a reliable for method for measurement of visceral fat’	Not specified	Yes	No
Bonjoch *et al*.	2022	Spain	Cross‐sectional, case‐control	20	Perirenal fat measured in the left posterior oblique position, by two technicians	‘Preliminary findings suggest that abdominal fat layers, especially subcutaneous and pre‐peritoneal fat, differ between people living with HIV and controls and are probably clinically relevant in terms of cardiovascular risk’	Two technicians	Yes	Yes
Borruel *et al*.	2012	Spain	Case‐control	99	Distance from the perirenal fascia to the renal surface on a long axis of the right kidney	‘…Ultrasound shows an excellent correlation with computed tomography … and magnetic resonance imaging …in the estimation of adipose tissue depots and correlates with cardiometabolic risk markers better than do anthropometric measurements also in patients with PCOS…’	Radiologist	Not stated	No
Borruel *et al*.	2014	Spain	Case‐control	86	Distance from the perirenal fascia to the renal surface on a long axis of the right kidney	‘In young adults, WC and BMI are the easiest to obtain and also the most accurate markers of visceral adiposity, as assessed by ultrasound, of the surrogate markers studied here. All the coefficients of correlation between WC, BMI and the thicknesses of abdominal adiposity were well‐above 0.5’	Radiologist	No	No
Carbone *et al*.	2018	Italy	Cohort revisit	110	PRAT measured posterior right renal wall (after Hirooka *et al*. 2005)	‘Ultrasound is not the gold standard method to measure visceral and subcutaneous fat areas as compared to computerised tomography and magnetic resonance. However, ultrasound is increasingly validating… whereas the other approaches are burdened by high costs and limitations due to the accessibility, contraindications and effect of radiation’	Not specified	No	No
Cirillo *et al*.	2021	Italy	Cross‐sectional	60	The thickness of fat (consisting of para‐ and perirenal fat) was measured from the inner side of the abdominal musculature to the surface of the kidney and designated ultrasound measure (UM) sonographic evaluation of visceral fat by measuring para‐ and perirenal fat. The average of the UM values on both sides was defined as the para‐ and perirenal ultrasound fat thickness	‘Our findings provided evidence of a significant correlation between para‐ perirenal fat and both lower HDL‐C and increased fasting plasma glucose, parameters defining metabolic syndrome’	Sonographer	Yes	No
D"Marco *et al*.	2019	Spain	Cohort	103	PRAT measured in the distal third (of the kidney) between the cortex and the hepatic border and/or spleen	‘…Our results revealed metabolic risk factors that correlated significantly with PRF thickness, which may affect kidney function. It remains to be determined whether there is a direct relationship between PRF and renal disease’	Not specified	No	Yes
Grima *et al*.	2009	Italy	Cohort	70	The visceral fat thickness was determined with a 3.75‐MHz convex transducer at a specific referee point as PRFT (longitudinal scan along the right mid‐clavicular line, from the border of the right liver lobe to the border of the inferior pole of the right kidney… with the patient in the supine position	‘…It could be useful to include PRFT/BMI measurement in screening evaluations of HIV‐1‐infected patients… to identify those at high risk of endothelial damage with development of atherosclerosis and cardiovascular diseases’	Trained sonographer	Yes	Yes
Grima *et al*.	2010	Italy	Cohort	88	PRFT (longitudinal scan along the right mid‐clavicular line, from the border of the right liver lobe to the border of the inferior pole of the right kidney… with the patient in the supine position	‘…Our data show that ultrasonographic measurement of PRFT could be considered a simple and objective parameter for early diagnosis of atherosclerosis. Furthermore, it allows identification of patients at increased risk of cardiovascular disease for which other clinical studies are useful’	Trained sonographer	Yes	Yes
Hazem *et al*.	2020	Saudi Arabia	Cross‐sectional	92	Posterior right perinephric fat thickness (PRPFT), the maximum fat thickness of the posterior right renal wall, was measured in the posterior right perinephric space	‘…Posterior perinephric fat thickness as measured via ultrasound was found to be a strong predictor of carotid atherosclerosis, followed by VFT/SCFT, VFT, VAT, PPFT, and WHtR with regard to predictive capacity. Additionally, posterior perinephric fat thickness was identified as a strong predictor for carotid artery plaque, followed by VFT/SCFT and the AFI’	Radiologist	Yes	Yes
Hazem *et al*.	2021	Saudi Arabia	Cross‐sectional	90	Posterior right perinephric fat thickness (PRPFT), that is the mean of the maximum fat thickness of the posterior right renal wall measured in the posterior right perinephric space (at three sites posterior to the upper, middle, and lower parts of the kidney)	‘Posterior perinephric fat thickness and visceral adipose tissue volume measured by ultrasound are strong non‐invasive predictors of coronary artery disease’	Experienced sonographer	Yes	Yes
Ke *et al*.	2021	China	Cross‐sectional	58	Para‐ and perirena fat thickness measured from inner surface of abdominal musculature to renal surface, both sides averaged, patient supine, using inspiration	This study showed that the renal adipose layers were ‘independently and negatively associated with the serum HDL level (and) independently and positively related (to) cholesterol efflux capacity’ and that further research on this subject was required ‘to illuminate it’	A single well‐trained operator	Yes	No
Manno *et al*.	2019	Italy	Cross‐sectional	102	The ultrasound thickness of para‐ and perirenal fat was measured from the inner side of the abdominal musculature to the surface of the kidney, and the average of the ultrasound measurement of the maximal thickness values on both sides was defined as the PUFT	‘Recent studies have also shown that epicardial fat accumulation is a risk factor for developing fibrillation and detrimental cardiovascular events… In conclusion, the present study… shows a direct independent relationship between epicardial fat and para‐ and perirenal fat measured by ultrasounds’	Sonographer	Yes	No
Morano *et al*.	2015	Italy	Cohort	25	The thickness of fat was measured from the inner side of the abdominal musculature to the surface of the kidney, and the average of both sides was defined as fat width at that site	‘…Visceral fat levels seem to predict the risk of cardiovascular disease better than crude anthropometric indexes as BMI or waist circumference…Interestingly, ultrasound proved to be a valuable tool to monitor treatment‐induced changes of adipose tissue storage’	Well‐trained sonographer	Yes	No
Okeahialam *et al*.	2020	Nigeria	Cross‐sectional	221	The PRF was measured using a 3.5 MHz probe. This is the echogenic strip between the posterior part of the liver and right kidney	‘PRF has shown to be correlated significantly with indices that predict atherosclerosis. Being an ectopic fat focus, its local and systemic effects on the kidney increase systemic vascular resistance and CVD (and) it can easily be measured on abdominal ultrasound’	Not specified	Yes	No
Ricci *et al*.	2018	Italy	Cohort	284	Measurement of perirenal fat thickness was taken from the right kidney lower pole to the psoas muscle surface	… ‘This study first identifies that the hypertension remission observed after bariatric surgery is related to perirenal fat thickness… we can state that the thickness of perirenal fat, evaluated by ultrasound method, could represent, in the obese subject, an integrated parameter able to define both the risk of developing arterial hypertension and chronic renal pathology’	Not specified	Yes	No
Roever *et al*.	2015	Brazil	Cross‐sectional	101	‘…The image of the right kidney and perirenal area was observed, with posterior measurement of the lateral hipechoic (sic) area that matches to the PRF in millimetres’	‘The perirenal ultrasonographic fat thickness measurement may… reflect the risks commonly associated with increased visceral fat accumulation and particularly those related to renal function impairment, microalbuminuria, hypertension, and uricaemia (and) may indicate individuals who have an increased atherosclerotic disease development potential’	Not specified	Yes	Yes
Sahin *et al*.	2014	Turkey	Case‐control	108	The combined thickness of para‐ and perirenal fat (PFT) was measured from the inner side of the abdominal musculature to the surface of the kidney	‘PFT values were higher particularly in non‐obese PCOS patients compared with non‐obese control subjects… We also observed a significant interaction between PCOS and obesity on PFT’	Radiologist	No	No
Shojaei *et al*.	2010	Iran	Cross‐sectional	106	The perirenal fat thickness was considered as the distance in millimetres between the lateral border of the kidney and the internal border of iliopsoas muscle surface adjacent to the middle third of the right kidney	‘Sonography is a reliable and available method for the estimation of body fat and its distribution in cardiovascular patients, in subcutaneous and visceral compartments’	Sonographer	No	No
Soattin *et al*.	2013	Italy	Cross‐sectional	105	Perirenal fat was determined as the distance between the kidney and the Gerota's fascia, and the para‐renal fat was calculated as the distance between the Gerota's fascia and the abdominal muscle	‘…Our data showed that only peritoneal fat thickness, among ultrasound parameters, is related to metabolic abnormalities after adjusting for BMI…the results of the present study suggest that SAD (sagittal abdominal diameter) and ultrasound peritoneal fat thickness may be the strongest predictors of visceral fat accumulation obesity’	Not specified	No	No
Topaloglu *et al*.	2014	Turkey	Cross‐sectional	55	Thickness of the fat layer of the posterior right renal wall in the right posterior perinephric space	‘VF (visceral fat) volume evaluated by ultrasound can be accepted as a cause of subclinical atherosclerosis in GH (growth hormone) deficient hypopituitary patients, particularly male (patients)’	Radiologist	Yes	No

### Risk of bias and quality assessment

Risk of bias was evaluated using the Downs and Black checklist^18^ of 27 questions assessing reporting, external validity, internal validity (bias and confounding) and power, awarding one point for each. The results are set out in Table 2. Studies that rated >26–27 were graded as excellent, 20–25 good, 15–19 as fair and <14 as poor (Page *et al*.^15^).

**Table 2:** Risk of bias assessment in the included studies using Downs and Black checklist (1998).

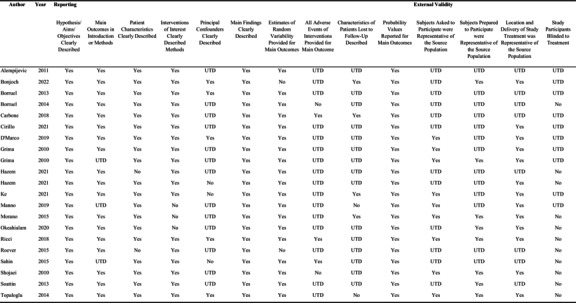
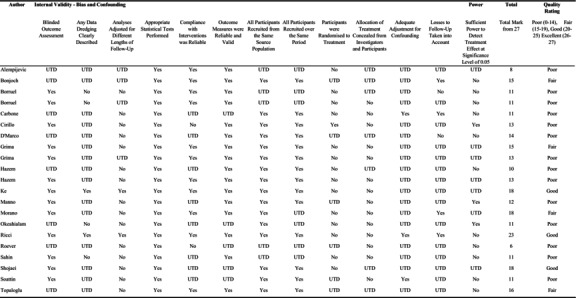

Abbreviation: UTD, unable to determine.

The Grade of Recommendations, Assessments, Development and Evaluation (GRADEpro)^19^ 2022 framework was used to evaluate the quality of the studies in terms of certainty of evidence, including risk of bias, inconsistency, indirectness, imprecision and publication bias. GRADE certainty of evidence is given in Table 3.

**Table 3:** GRADE certainty of evidence. Question: Ultrasound of peri renal adipose tissue compared to not using ultrasound for indicating cardiovascular disease in at‐risk individuals. Author(s): Baumann and Clarke. Setting: screening.

Certainty assessment (assessed from 21 included studies)							Comment	Certainty	Importance
No. of studies	Study design	Risk of bias	Inconsistency	Indirectness	Imprecision	Other considerations			
**Reproducibility**									
19	Non‐randomised studies	Serious^a^	Serious^a^	Not serious	Not serious	None	Result: 89% of included studies found ultrasound measurement of PRAT to be reproducible among practitioners, regardless of whether a trained sonographer or a Radiologist performed the scan. Note: This systematic review included 2053 patients in total: 1896 subjects and 177 controls across the various studies. Reproducibility was not measured in two studies, including 128 patients and 40 controls	⨁⨁◯◯ Low	CRITICAL
**Reliability**									
20	Non‐randomised studies	Serious^a^	Not serious^a^	Not serious	Not serious	None	Result: 89% of studies found ultrasound measurement of PRAT to be reliable as an index for fat indices. Note: Two studies did not address reliability, this involved 385 patients	⨁◯◯◯ Very low	CRITICAL
**Predictive of cardiovascular disease**									
21	Non‐randomised studies	Not serious	Not serious	Not serious	Not serious	None^b^	Result: 100% of studies found ultrasound measurement of PRAT thickness to be predictive of CVD or metabolic disease. Note: This involved 2053 subjects, including 1896 patients and 177 controls	⨁⨁◯◯ Low	CRITICAL
**Accuracy compared to other measurements and imaging modalities**									
15	Non‐randomised studies	Not serious	Serious^c^	Not serious	Not serious	Strong association between assessment of certainty and accuracy of US compared to CT and MRI	Result: 79% of studies found ultrasound measurement of PRAT thickness to be accurate compared to CT and MRI. Notes: Six studies did not state accuracy of ultrasound compared to CT and MRI. This involved 532 subjects: 560 patients and 50 controls	⨁⨁◯◯ Low	CRITICAL
**Variability in ultrasound techniques**									
19	Non‐randomised studies	Serious	Very serious^a^	Very serious^a^	Serious^a^	Very strong association between assessment of certainty and US measurement technique	Result: 90% variability in measurement technique. Note: Variability in technique for measuring PRAT thickness with ultrasound was demonstrated in 19 out of 21 studies, involving 2053 subjects	⨁◯◯◯ Very low	CRITICAL

^a^ No standardised guidelines evident in the ultrasound measurement of PRAT.

^b^ All studies showed ultrasound measurement to be predictive of metabolic disease.

^c^ Four studies did not discuss accuracy of ultrasound compared to CT or MRI.

## Results

The evaluation of the published data consisted of summarising the characteristics and quality of the included studies, and the techniques employed to measure PRAT thickness. Outcomes using ultrasound as an indicator of CVD were assessed in terms of reliability and reproducibility, correlation with metabolic factors, predictive capability, accuracy compared with MRI and CT imaging and variations in ultrasound technique. The characteristics of the included studies are set out in Table 1. The patient population included those at risk for CVD, who had ultrasound measurement of PRAT and the ultrasound features of this fat.

### Ultrasound techniques

This review identified great variation in both ultrasound techniques and resulting measurements. Twenty different techniques were described over the 21 studies, making it clear there are no standardised guidelines for this measurement. Notably 10 of these studies stipulated using the measurement from the right perinephric area.^2^, ^5^, ^13^, ^20^, ^21^, ^22^, ^23^, ^24^, ^25^, ^26^ Supine patient positioning was used in 11 studies,^5^, ^22^, ^23^, ^24^, ^25^, ^27^, ^28^, ^29^, ^30^, ^31^, ^32^, ^33^, ^34^, ^35^ and light transducer pressure to minimise compression of the fat layer was mentioned in nine studies.^5^, ^22^, ^23^, ^24^, ^26^, ^27^, ^29^, ^30^, ^34^, ^36^ Respiration was arrested in eight studies,^23^, ^25^, ^26^, ^29^, ^30^, ^31^, ^34^, ^35^ but in three different phases. Fasting was used in two studies.^21^, ^33^ Further, six studies^24^, ^27^, ^28^, ^31^, ^33^, ^35^ measured the fat thickness from the inner surface of the abdominal musculature to the renal surface bilaterally and averaged the findings to create a PRAT measurement. Qualifications and experience of the ultrasound operators were either not standardised or not mentioned. The remainder of the studies all had different measurement methods. These findings are set out in Table 4 (Summary of techniques for ultrasound measurement of PRAT).

**Table 4:** Summary of ultrasound techniques for measuring PRAT.

Authors	Technique
Alempijevic *et al*. (2011), Borruel *et al*. (2013, 2014), Cirillo *et al*. (2021), Grima *et al*. (2010, 2010), Hazem *et al*. (2020, 2020), Ke *et al*. (2021), Manno *et al*. (2019), Sahin *et al*. (2014), Soattin *et al*. (2013) and Topaloglu *et al*. (2014)	Supine position
Bonjoch *et al*. (2022) and Roever *et al*. (2015)	Dorsal decubitus position
Alempijevic *et al*. (2011), Borruel *et al*. (2013, 2014), Cirillo *et al*. (2021), Grima *et al*. (2010, 2010), Hazem *et al*. (2020, 2020) and Manno *et al*. (2019)	Light transducer pressure
Manno *et al*. (2019), Morano *et al*. (2015), Shojaei *et al*. (2010), and Soattin *et al*. (2013)	Longitudinal scan with kidney parallel to skin, or scanning in the mid‐clavicular line
Alempijevic *et al*. (2011), Borruel *et al*. (2010, 2014), Hazem *et al*. (2020, 2020), Ke *et al*. (2021), Morano *et al*. (2015) and Topaloglu *et al*. (2014)	Arrested respiration: any phase
Borruel *et al*. (2013, 2014)	Perirenal space: fascia to kidney surface
Alempijevic *et al*. (2011)	Measurement of posterior peri‐nephric space
Manno *et al*. (2019), Ricci *et al*. (2018) and Roever *et al*. (2015)	Right perirenal area
D'Marco *et al*. (2019) and Ricci *et al*. (2018)	Distal third of renal cortex to hepatic/splenic border
D'Marco *et al*. (2019) and Ricci *et al*. (2018)	Distal third of (right) renal cortex to the right psoas muscle
Shojaei *et al*. (2010)	Lateral middle third of kidney to internal border of iliopsoas
Grima *et al*. (2010, 2010)	Mid‐clavicular line, right lobe of liver to inferior border of right kidney
Grima *et al*. (2010, 2010)	Triple determination of measurement
Hazem *et al*. (2020, 2020)	Maximum fat thickness of the right perinephric space
Cirillo *et al*. (2021), Ke *et al*. (2021), Manno *et al*. (2019), Morano *et al*. (2015), Sahin *et al*. (2014) and Soattin *et al*. (2013)	Inner surface of abdominal musculature to renal surface, both sides averaged
Okeahialam *et al*. (2020)	Echogenic strip between posterior part of the liver and right kidney
Topaloglu *et al*. (2014)	Left PRAT: Internal surface of splenic vein to abdominal musculature
Bonjoch *et al*. (2022) and Borruel *et al*. (2013)	Two technicians/sonographers performed the measurements
Topaloglu *et al*. (2014)	Right PRAT: thickness of fat layer in right posterior perinephric space
D'Marco *et al*. (2019) and Soattin *et al*. (2013)	Fasting state

### Outcomes of PRAT thickness as an indicator of CVD

#### Reliability and reproducibility

As set out in Table 3, all authors found the ultrasound measurement of PRAT thickness to be predictive of CVD or metabolic disease, however with low certainty on the GRADE certainty of evidence scale. This implies that further research is likely to change the estimate. Nearly all studies, 20 out of the 21, found this measurement to be reliable, albeit with very low certainty of effect and reproducibility. Ricci^13^ and Roever^20^ investigated risk factors linking CVD with PRAT but did not mention reliability; Cirillo^27^ and Sahin^28^ studied women considering assisted reproduction or those with polycystic ovarian syndrome (PCOS) and PRAT thickness but did not mention reproducibility. Overall, regarding reliability and reproducibility, the quality of the studies was poor.

#### Correlation with metabolic factors

Perirenal adipose tissue thickness is strongly tied to metabolic syndrome, with its cluster of factors including increased waist circumference, hypertension, insulin resistance, high triglyceride levels and low‐ to high‐density lipoprotein cholesterol.

Alemoijevic *et al*.^29^ found PRAT predictive of morbidity, including atherosclerosis and metabolic syndrome. Borruel *et al*.^30^ found the measurement of organ‐specific visceral adipose tissue showed positive correlations with blood pressure, insulin and cholesterol levels. One study, by D'Marco *et al*.^21^ demonstrated that patients with chronic renal disease are at a greater risk for cardiovascular events, hypertension, left ventricular hypertrophy and coronary artery disease than other at‐risk groups. They concluded perirenal fat thickness correlated significantly with metabolic risk factors, which could affect renal function. A combined measurement of both para‐ and perirenal fat thicknesses was found by Ke *et al*.^31^ to be ‘closely correlated with serum high density lipoprotein and cholesterol efflux capacity in patients with Type 2 Diabetes Mellitus (T2DM)’.

Shojaei *et al*.^32^ sought to use ultrasound to evaluate whole body fat volume and separately measured subcutaneous and visceral fat in their study of metabolic syndrome. Soattin *et al*.^33^ assessed anthropometry, sonography and abdominal bio‐electrical impedance as predictors of metabolic abnormalities in both normal and obese patients, in a detailed study measuring perirenal, pararenal, perihepatic, preperitoneal and peritoneal fat thicknesses. Their results suggested that sagittal abdominal diameter and ultrasound‐determined perirenal fat thickness may be the strongest predictors of visceral fat accumulation, obesity and its related metabolic abnormalities, irrespective of age, sex and the degree of obesity. They found ultrasound to be reliable, predictive, reproducible and accurate compared with other imaging methods.

#### Outcomes related to accuracy of ultrasound

Eight studies (79%) reported ultrasound measurement of PRAT thickness to be accurate compared with CT and MRI. For example, Borruel *et al*.^34^ sought to find an objective, relatively inexpensive and radiation‐free method to measure visceral adiposity using ultrasound, finding moderate correlation between it and other measurements such as waist circumference and BMI and excellent correlations with CT and MRI.

#### Correlation between PRAT and carotid intima thickness

Carbone *et al*.^36^ found ultrasound to be ‘increasingly validating’ for measuring PRAT and Ricci^13^ reported ultrasound to be predictive, reproducible and accurate compared with other imaging modalities. The Uberlandia Heart Study^20^ found ultrasound both reproducible and predictive for CVD.

Grima *et al*.^5^, ^22^ studied visceral fat accumulation as a risk factor for atherosclerosis, in human immunodeficiency virus (HIV) patients, and determined carotid intima thickness was related to PRAT.

Hazem *et al*.^23^ looked at ultrasound measurements of multiple abdominal fat deposits for predicting the presence and severity of carotid artery disease, concluding that posterior perinephric fat thickness together with visceral adipose tissue (VAT) volume are strong non‐invasive predictors for coronary artery disease, followed by BMI, waist circumference and visceral fat thickness (VFT).

Okeahialam *et al*.^2^ found relationships between PRAT measured by ultrasound and subclinical atherosclerosis, indexed by carotid intima‐media thickness and other indirect measures of cardiometabolic disease risk. These authors referenced the work by Fatkhllina *et al*.^37^ who discussed the presence of low‐grade inflammation concurrent with high VAT and inflamed fat‐producing cytokines, which promote atherosclerosis.

#### Outcomes using ultrasound as an indicator of CVD

A direct independent and previously unrecognised relationship between epicardial fat and para‐ and perirenal fat, measured with ultrasound, was found by Manno *et al*.^24^ The authors concluded that the adipocytokines produced by epicardial fat possibly induce local, coronary and heart effects and that those produced by para‐ and perirenal fat may be more likely to induce systemic effects. They also suggested that an increase in PRAT is independently associated with insulin resistance and cholesterol changes.

Morano *et al*.^35^ used ultrasound to assess the effects of a trial drug (glucagon‐like peptide 1) on fat distribution of patients with T2DM. Ultrasound was used to measure subcutaneous fat, both periumbilical and subxiphoid; deep fat deposits, including preaortic, perirenal and epicardial fats; and the renal resistive index, enabling the researchers to assess a distribution of adipose tissue deposits, which possibly contributed to an improved cardiovascular risk profile in their patients with T2DM.

In the study by Ricci *et al*.^13^ the significant difference in PRAT thickness between male and female obese patients led them to conclude that ultrasound measurements of PRAT could provide ‘an integrated parameter to define both the risk of developing arterial hypertension and chronic renal pathology’ (p. 1436). Further, they believed this could define those who need more aggressive treatment including bariatric surgery. Sex‐specific differences also featured in the correlation of CVD risk factors and PRAT assessed with ultrasound in a Brazilian population by Roever *et al*.^20^ Conversely, the study by Shojaei *et al*.^32^ did not find significant sex differences in sonographic predictions of subcutaneous and visceral, including perirenal, fat mass.

Assessing male patients with hypopituitarism and growth hormone (GH) deficiency, researchers Topaloglu *et al*.^25^ found GH‐deficient hypopituitary patients, with or without sex steroid deficiencies, had increased visceral fat (VF) compared with healthy controls, and confirmed ‘the usefulness’ of ultrasonic measurement of VF volume. Their results indicate that excessive VF accumulation affects the growth of subclinical atherosclerosis in GH‐deficient men.

The results of Bonjoch *et al*.^38^ suggested ‘the thickness of abdominal fat layers, assessed by ultrasound could be a marker of cardiovascular risk’ (p. 222). Due to their small sample size, they recommended further studies on larger populations to confirm their findings. Table 5 details further findings, and the self‐reported limitations of the included studies.

**Table 5:** Summary of findings and limitations included studies (n = 21).

Author and year	Study type	Number of participants	Downs and Black quality assessment	Main findings	Limitations
Alempjevic *et al*. (2011)	Case control	90	Poor	Ultrasound is reliable for measurement visceral fat and could be as effective as CT	Not mentioned
Bonjoch *et al*. (2022)	Cross section	10	Poor	The thickness of abdominal fat layers, assessed by ultrasound, could be a marker of cardiovascular risk	Further studies with larger populations are required to confirm their findings
Bonjoch *et al*. (2022)	Cross section and case control	20	Fair	Ultrasound is an inexpensive, non‐invasive tool that enables (measurement of) abdominal fat layers as easily detectable markers and thus identify persons at high risk of cardiovascular disease	Small sample size
Burruel *et al*. (2013)	Case control	106	Poor	Women with PCOS have higher global adiposity and increased VAT compared with controls, measured by ultrasound and including PRAT	Ultrasound the only modality used. Not all fat deposits measured
Burruel *et al*. (2014)	Case control	99	Poor	WC and BMI are the easiest markers of visceral adiposity to obtain, of the markers assessed here, assessed by ultrasound	Not mentioned
Carbone *et al*. (2018)	Cohort	110	Poor	High sensitivity C‐reactive protein might be a promising marker of VAT fat loss in obese females after sleeve gastrectomy. Measured according to the Hirooka's formula (2005), which includes the thickness of the fat layer of the posterior right renal wall	Subanalysis of a previous study, hence potential biases. Small sample size. Ultrasound not the gold standard (CT and MRI are.). Study design did not include sarcopenic obesity
Cirillo *et al*. (2021)	Cross section	60	Poor	Highlights a ‘dangerous liaison’ between ectopic fat deposits and metabolic/inflammatory markers. Sonographic evaluation of visceral fat by measuring para‐ and peri renal fat thickness (as per Kawasaki 2008)	Selection bias: no measurement of normal weight women, only Caucasian women (fat distribution may vary according to race/ethnicity in reproductive aged women)
D'Marco *et al*. (2019)	Cohort	103	Poor	In CRD patients, the PRAT thickness correlated significantly with metabolic risk factors, which could affect kidney function	Small sample size. Lack if data on additional metabolic and inflammatory biomarkers (C‐reactive protein, cystatin C and asymmetric dimethylarginine levels)
Grima *et al*. (2009)	Cohort	70	Fair	Ultrasound measurement of PRAT could be used as an early predictor of intima‐media thicknesses in HIV‐infected patients receiving highly active antiretroviral therapy	Small sample size
Grima *et al*. (2010)	Cohort	88	Fair	Ultrasound assessment of PRAT may have potential as marker of increased endothelial damage with specific involvement of the ocular vascular region in HIV‐infected pts	Limitations of ultrasound assessment, including operator pressure, presence of bloating, poor accuracy for obese patients
Hazem *et al*. (2020)	Cross section	92	Poor	Many abdominal fat indices, including ultrasound assessed PRAT thickness, are strong predictors of carotid artery plaque and atherosclerosis	Small sample size. Possible selection bias as all subjects drawn from internal medicine clinics. Clinical overlap possible.
Hazem *et al*. (2021)	Cross section(revisit)	90	Poor	Posterior PRAT and VAT volume, measured by Ultrasound, are strong non‐invasive predictors for coronary artery disease, followed by BMI WC and VFT	Small sample size. Possible selection bias as all subjects drawn from internal medicine clinics. Clinical overlap possible. High prevalence of medication in this group likely affected correlations with coronary artery disease
Ke *et al*. (2021)	Cross section	58	Fair	Para‐ and perirenal ultrasonographic fat thickness is closely correlated with serum HDL level and cholesterol efflux capacity in patients with T2DM	A cross‐sectional study could not establish the causal relation between renal fat thickness and HDL levels and cholesterol efflux capacity. Possible variation in measurement, using ultrasound instead of CT, was controlled by using ‘one well‐trained operator’
Manno *et al*. (2019)	Cross section	102	Poor	Increased central fat in apparently healthy overweight patients is associated with similar increases in para renal, perirenal and epicardial fat, measured using ultrasound. A direct relationship shown between epicardial fat and para/perirenal fat thicknesses	Small sample size. Study design error: a control group could have reinforced the relevance of the findings
Morano *et al*. (2015)	Cohort	25	Fair	A short course of treatment with GLP‐1 RA, besides weight loss, induces a redistribution of adipose tissue deposits, possibly contributing to a better cardiovascular risk. Measurements included ultrasound measurement of PRAT. profile in patients with type 2 diabetes mellitus	Small sample size
Okeahialam *et al*. (2020)	Cross section	221	Poor	PRAT thickness, measured with ultrasound, was shown to be correlated significantly with indices that predict atherosclerosis. Being an ectopic fat focus, its local and systemic effects on the kidney increase systemic vascular resistance and CVD	Selection bias: single‐centre and single‐race study
Ricci *et al*. (2018)	Cohort	284	Good	PRAT thickness in obese patients, measured with ultrasound, could be a valuable tool to define the risk of developing hypertension, providing the clinician with an additional parameter to define those who need a more aggressive treatment and could benefit most from bariatric surgery	Interventional study design prevents drawing a conclusion on causative effect of PRAAT thickness on hypertension development in obese patients. The absence of a control groups precludes comparison between sleeve‐gastrectomy intervention with other weight loss strategies. Short follow‐up time, so study will be revisited. Small sample size
Roever *et al*. (2015)	Cross section	101	Poor	PRAT thickness, measured with ultrasound, was correlated with most cardiovascular risk factors in men and only in glucose at the women	None mentioned.
Sahin *et al*. (2014)	Case control	68	Poor	PRAT thickness values, measured with ultrasound, were higher, particularly in non‐obese PCOS patients, compared with non‐obese control subjects. There was a significant interaction between PCOS and obesity on PRAT thickness	Small sample size. Total testosterone levels not measured with gold standard assays
Shojaei *et al*. (2010)	Cross section	106	Poor	Sonography is a reliable and available method for the estimation of body fat and its distribution in cardiovascular patients, in subcutaneous and visceral compartments	Limitations of BMI and ultrasound measurement, not of the study itself. Ultrasound is highly operator dependent, requiring a thorough knowledge of anatomy and techniques
Soattin *et al*. (2013)	Cross section	Cross section 105	Poor	The results of the present study suggest that SAD and ultrasound peritoneal fat thickness may be the strongest predictors of visceral fat accumulation obesity and its related metabolic abnormalities irrespective of age, sex and the degree of obesity	Ultrasound has a limited field of vision, no comparison with CT or MRI imaging
Topaloglu *et al*. (2014)	Cross section	55	Fair	VF volume, evaluated by ultrasound, can be accepted as a cause of subclinical atherosclerosis in growth hormone‐deficient hypopituitary patients, particularly males	Small sample size. Some confusion between terms perinephric and paranephric fat, possible due to translation

## Discussion

This systematic review presents a comprehensive narrative analysis of the current use of ultrasound measurements of PRAT for the prediction of CVD.

### Variation in measurement protocols

Significantly, 20 different techniques were used across 21 studies. Some studies^13^, ^20^, ^24^ report measurements of the right perirenal space or fat thickness. Others^30^, ^34^ report right perirenal fascia to renal surface. The average bilateral measurement was reported by six studies.^24^, ^27^, ^28^, ^31^, ^33^, ^35^ The issue of which technique for PRAT measurements is best represents an area for further research but has not been addressed in this review.

Acknowledging the wide range of populations across the studies, the wide range of values, 6.3–20.0 mm, may be partly explained by the wide variation in measurement techniques. For example, the study that yielded 20 mm included both para‐ and perirenal fat.^24^ Some studies involved patients with obesity issues^13^, ^24^, ^33^, ^36^ and others involved those with chronic disease.^5^, ^21^, ^22^, ^25^, ^29^, ^30^, ^34^, ^35^

Okeahialam *et al*.^2^ addressed the issue of non‐differentiation between fat and whole‐body mass, when only BMI is used. This was also raised by Shojaei *et al*.^32^ who concluded that the estimation of body composition and fat distribution by a method other than BMI would ‘be a great help’ (p. 85) in the prediction of CVD.

### Qualification of ultrasound operator

The experience and qualifications of the ultrasound operator was not well‐reported, despite the understanding that ultrasound techniques are highly operator‐dependent. Radiologists performed the scans in 11 studies,^5^, ^22^, ^23^, ^25^, ^26^, ^27^, ^28^, ^30^, ^32^, ^34^, ^35^ one study mentioned that scanning was performed by a sonographer,^24^ but many authors did not provide details.^2^, ^13^, ^20^, ^21^, ^29^, ^33^, ^36^, ^38^ Borruel *et al*.^30^ used two ‘technicians’ to enhance the reproducibility of their measurements. One study concluded ultrasound was a practical and effective modality for measuring body fat and its distribution, but the operator dependency meant operators must be thoroughly trained in anatomy and technique.^32^

Twenty studies were found to be reliable, assessed according to GRADE (Table 3).^19^ PRAT measurement by ultrasound was found to be predictive for CVD in all 21 studies; 17 studies found ultrasound results reproducible.^2^, ^5^, ^13^, ^21^, ^22^, ^23^, ^24^, ^25^, ^26^, ^27^, ^29^, ^30^, ^32^, ^34^, ^35^, ^36^, ^38^ Finally, 13 studies found ultrasound comparable in terms of accuracy to CT and MRI.^5^, ^21^, ^22^, ^23^, ^25^, ^26^, ^28^, ^29^, ^30^, ^32^, ^33^, ^34^, ^36^

### Links to epicardial fat

Perirenal adipose tissue thickness linked to epicardial adipose tissue is discussed by three studies. Manno *et al*.^24^ used ultrasound to show a direct relationship between para‐ and perirenal fat and epicardial fat. Morano *et al*.^35^ similarly assessed deep fat deposits, which included PRAT and epicardial fat, and felt these to be more predictive of CVD risk than other (fat) sites. Borruel *et al*.^30^ measured several fat deposits in their assessment of global adiposity in women with PCOS, including PRAT and epicardial fat.

### Results relating to sex

There were conflicting results for sex‐based differences in PRAT thickness. Shojaei *et al*.^32^ found sex differences not significant and felt that sonographic prediction was valid for both sexes. Despite small numbers, Ricci *et al*.^13^ found PRAT significantly different between sexes and concluded ‘this topic deserves further targeted studies’ (p. 1436). Roever *et al*.^20^ reported correlations between PRAT and most cardiovascular risk factors in men, ‘and with the levels of fasting plasma glucose in women’, (p. 4) concluding PRAT ‘is associated with an adverse metabolic risk profile’. Some studied a sex‐specific pathology such as PCOS^28^, ^30^ and those planning assisted reproduction^27^ or performed their study on a single‐sex population.^25^ Borruel *et al*.^30^ found ‘obesity increased the thickness of all the adipose tissue deposits’ in women with PCOS (p. 1254) and confirmed sexual dysmorphism in global fat distribution.

Sahin *et al*.^28^ found PCOS is associated with an increased risk of hypertension and CVD and a ‘significant’ interaction between PCOS and obesity.

### Considerations regarding ethnicity

According to the Harvard School of Public Health, Asians who have a 3–5% higher total body fat than White Europeans with the same BMI are more prone to developing obesity and have a high risk of T2DM and CVD.^39^ Two studies included in this review specified the ethnic make‐up of their subjects. Okeahialam *et al*.^2^ noted a limitation of their study was that it was a single‐centre study, in this case Nigeria, ‘which affects the generalization of findings’ (p. 567). Roever *et al*.^20^ designed their study to specifically gather missing data relevant to the Brazilian population, as ‘gender differences in the correlations of (CVD) risk factors and PRF in the Brazilian population are lacking’ (p. 1). Neither study addressed whether their data would translocate to a worldwide demographic. No other studies stipulated the ethnicity of their subjects, merely their nationality.

### Correlation between PRAT and carotid intima thickness

Finding BMI does not differentiate between fat and whole‐body mass, Okeahialam *et al*.^2^ looked for a more precise indicator of CVD risk. They investigated a possible link between PRAT thickness and carotid intima media thickness (CIMT) and found PRAT thickness ‘corresponded significantly with indices that predict atherosclerosis’. They concluded PRAT thickness >0.25 cm should prompt ‘initiation of action to prevent or mitigate atherosclerotic CVD’ (p. 567).

Grima *et al*.^22^ sought to study central obesity as an index of atherosclerosis in HIV‐1‐infected patients receiving antiretroviral therapy. These patients commonly show changes in their body fat distribution, called lipodystrophy syndrome. Researchers sonographically measured PRAT and correlated it with CIMT in a 12‐month cohort study. Since carotid wall‐thickening can reflect preclinical atherosclerosis at other sites, they considered PRAT thickness could be an indicator of CIMT increase in their patient cohort. In a second study in 2010,^5^ this team found increased PRAT thickness could indicate endothelial damage with specific involvement of the ophthalmic artery, in similar patients.

### Further implications

The close proximity of PRAT to the renal parenchyma and renal sinus and being bound by tight Gerota's fascia, means it can have a significant local and systemic mechanical effect.

D'Marco *et al*.^21^ found metabolic risk factors correlated significantly with perirenal fat thickness and suggested that further research is needed into the link between PRAT and renal disease. Compression of the parenchyma and renal arteries can directly lead to renal damage and increased systemic vascular resistance.^2^ This is an important finding that requires further research.

Morano *et al*.^35^ felt ‘the implementation of carefully standardized ultrasound measurement protocols is critical to obtain accurate and reliable results…’ (p. 730). An important finding of this review has been the lack of standardisation of ultrasound measurement of PRAT, and the technique for this will be addressed in a subsequent paper.

Further, more in‐depth studies are needed to elaborate race/ethnic and sex differences in body composition and how these differences relate to clinically meaningful risks.^40^ This is an area where investigation of PRAT and epicardial fat correlation is important and is an area for further research.

Incidentally, this review found further implications as a 2019 study^41^ found PRAT a reservoir of mesenchymal cells with the capacity to differentiate into adipocyte, osteogenic, chondrogenic and epithelial cells. Additionally, Lee *et al*.^42^ propose using donor perirenal fat, with its proliferation of metabolically active brown adipocytes, as ‘an effective human cell source… to treat metabolic diseases through energy consumption, rather than it being incinerated as medical waste’.

### Limitations

The main limitations of this review are the relatively small number of appropriate studies with no RCTs, lowering the certainty of evidence.

## Conclusion

We have shown that PRAT thickness measured by ultrasound merits further study as it is known to be associated with a high cardiovascular and/or metabolic risk.^4^, ^13^ Researchers are measuring PRAT with a variety of techniques and hence, finding variable results. Establishment of normal and abnormal values is particularly important. The development and adoption of a standardised protocol, which is efficient and easily incorporated into a routine abdominal ultrasound study, would be immensely beneficial.

In vivo, the most accurate measurement of PRAT is done with radiology: ultrasound, magnetic resonance imaging and computed tomography. Ultrasound is portable, cheap, fast and well‐tolerated by patients, involves no ionising radiation and is available at many sites where CT and MRI are not. It is non‐invasive and well‐suited for measuring PRAT. The transportable nature of ultrasound is a distinct advantage, particularly in rural and remote locations.

This review demonstrates the strong potential to move the measurement of PRAT from a research platform to clinical implementation. This would require standardised measurement guidelines and technique and importantly, education for users.

Appropriate training of sonographers and radiologists and further studies involving a standardised measure of PRAT, will add a valuable tool, particularly where high‐end modalities are unavailable. The readily available ultrasound measurement of PRAT should prompt initiation of preventive or curative intervention where it is found to be abnormally thick.

### Registration

According to PRISMA Guidelines: Preferred Reporting Items for Systematic Reviews and Meta Analyses (PRISMA Group 2009), this systematic review was designed a priori.

It was registered with the International Prospective register of Systematic Reviews (PROSPERO) on 15 January 2022 (CRD42022293769), under the title *What features of perirenal adipose tissue seen on ultrasound indicate cardiovascular disease?*

## Authorship statement

We acknowledge that (i) the authorship listing conforms with the journal's authorship policy, and (ii) that all authors are in agreement with the content of the submitted manuscript.

## Funding

The authors received no funding in the production of this systematic review.

## Conflict of interest

We declare we have no funding support or relationships, which may pose any conflicts of interest.

## Supporting information


**Appendix S1** Search terms.

**Appendix S2** Summary of excluded studies (n = 141).
