# Supplementary material for: Ultrasound measurement of perirenal adipose tissue indicates cardiovascular disease, but standardisation is needed: A systematic review
Source: Australas J Ultrasound Med. 2024 Oct 20;28(1):e12407. doi: 10.1002/ajum.12407 (PMC11761460; doi:10.1002/ajum.12407)
Supplement: Supplementary file 1 — Appendix S1 Search terms. [file AJUM-28-0-s001.pdf]

Appendix 1: Search terms

Abdominal fat
Adherent perinephric fat
Adipokines
Adiponectin
Adipose tissue
Adipose tissue and diagnostic imaging
Brite fat
Brown adipose tissue
Brown fat
Cardiovascular diseases
Cardiovascular risk
CKD chronic kidney disease
CRD chronic renal disease
Ectopic brown fat
End stage renal disease
Fat-kidney interaction
Glomerular filtration rate
hypertension
Imaging
Kidney
Kidney fat
Metabolic disease
Metabolic syndrome
Obesity
Pararenal fat
Pararenal fat mass
Pararenal fat thickness
Pararenal fat volume
Peri organ fat deposits
Perirenal adiposity
Perirenal fat
Perirenal fat mass
Perirenal fat thickness
Perirenal fat volume
Proinflammatory cytokines
Renal
Renal fat pad
Renal ultrasound
Renin-angiotensin system
retroperitoneum
Systemic disease
Ultrasound
Visceral adipose tissue
Visceral fat
White fat
