# Supplementary material for: Ultrasound measurement of perirenal adipose tissue indicates cardiovascular disease, but standardisation is needed: A systematic review
Source: Australas J Ultrasound Med. 2024 Oct 20;28(1):e12407. doi: 10.1002/ajum.12407 (PMC11761460; doi:10.1002/ajum.12407)
Supplement: Supplementary file 2 — Appendix S2 Summary of excluded studies (n = 141). [file AJUM-28-0-s002.pdf]

Appendix 2: Summary of excluded studies (n=141)

Author		Article title	Reason for exclusion
Ali, M. M.; Naquiallah, D.; Qureshi, M.; Mirza, M. I.; Hassan, C.; Masrur, M.; Bianco, F. M.; Frederick, P.; Cristoforo, G. P.; Gangemi, A.; Phillips, S. A.; Mahmoud, A. M.	2022	DNA methylation profile of genes involved in inflammation and autoimmunity correlates with vascular function in morbidly obese adults	PRAT not specifically measured
Al-Mallah, M. H.; Lloyd, S. G.; Doukky, R.; AlJaroudi, W. A.; Hage, F. G.	2019	Multi-modality imaging: Bird's eye view from the 2018 American Heart Association Scientific Sessions	Editorial
Andrew Ryabikov, A.; Guliev, Z.; Malyutina, S.	2013	Novel ultrasound screening approach for subcutaneous and abdominal fat measurement	Study not able to be retrieved
Angoorani, H.; Karimi, Z.; Naderi, F.; Mazaherinezhad, A.	2018	Is ultrasound-measured abdominal fat thickness a reliable method for predicting metabolic diseases in obese and overweight women?	PRAT not specifically measured
Antonopoulos, A. S.; Herdman, L.; Thomas, S.; Akoumianakis, I.; Kotanidis, C.; Thomas, K.; Oikonomou, E. K.; Psarros, K.; Sayeed, R.; Antoniades, C.	2018	Metabolically healthy obesity is associated with a distinct epicardial fat phenotype and low myocardial oxidative stress	Conference paper
Arepalli, C. D.; Vrettoy, R. A.; Lamanna, J. J.; Ebert, E. L.; Kremastinos, D. T.; Lerakis, S.	2012	Epicardial and pericardial adipose tissue: physiological importance and role of imaging techniques	PRAT not specifically measured
Azzi, A. J.; Lafreniere, A. S.; Gilardino, M.; Hemmerling, T.	2019	Ultrasonography Technique in Abdominal Subcutaneous Adipose Tissue Measurement: A Systematic Review	Literature or systematic review
Baranauskas, M. N.; Johnson, K. E.; Juvancic-Heltzel, J. A.; Kappler, R. M.; Richardson, L.; Jamieson, S.; Otterstetter, R.	2017	Seven-site versus three-site method of body composition using BodyMetrix ultrasound compared to dual-energy X-ray absorptiometry	PRAT not specifically measured
Basheer, M.; Saad, E.; Jeries, H.; Assy, N.	2023	Liver Fat Storage Is a Better Predictor of Coronary Artery Disease than Visceral Fat	Erroneous study design
Bazzocchi, A.; Filonzi, G.; Ponti, F.; Albisinni, U.; Guglielmi, G.; Battista, G.	2016	Ultrasound: Which role in body composition?	Literature or systematic review
Bazzocchi, A.; Filonzi, G.; Ponti, F.; Sassi, C.; Salizzoni, E.; Battista, G.; Canini, R.	2011	Accuracy, Reproducibility and Repeatability of Ultrasonography in the Assessment of Abdominal Adiposity	PRAT not specifically measured
Bazzocchi, A.; Filonzi, G.; Ponti, F.; Sassi, C.; Salizzoni, E.; Battista, G.; Canini, R.	2011	Accuracy, reproducibility and repeatability of ultrasonography in the assessment of abdominal adiposity	Editorial

Bellan, M.; Menegatti, M.; Ferrari, C.; Carnevale Schianca, G. P.; Pirisi, M.	2018	Ultrasound-assessed visceral fat and associations with glucose homeostasis and cardiovascular risk in clinical practice	PRAT not specifically measured
Bellisari, A.; Roche, A. F.; Siervogel, R. M.	1993	Reliability of B-mode ultrasonic measurements of subcutaneous adipose tissue and intra-abdominal depth: comparisons with skinfold thicknesses	Study outside criteria dates
Bemelmans, R. H.; van der Graaf, Y.; Nathoe, H. M.; Wassink, A. M.; Vernooij, J. W.; Spiering, W.; Visseren, F. L.; Smart Study	2012	Increased visceral adipose tissue is associated with increased resting heart rate in patients with manifest vascular disease	
Benevides,F.T.;Araujo Junior,E.;Maia,C,S,C.;Maia e Holanda Moura,S.B.; Montenegro Junior,R,M.;Carvalho,F,H,C.	2022	Evaluatioon of ultrasound measurements of abdominal fat for the prediction of gestational diabetes in the first and second trimesters of pregnancy	PRAT not specifically measured
Berker, D.; Koparal, S.; Isik, S.; Pasaoglu, L.; Aydin, Y.; Erol, K.; Delibasi, T.; Guler, S.	2010	Compatibility of different methods for the measurement of visceral fat in different body mass index strata	PRAT not specifically measured
Bertaso, A. G.; Bertol, D.; Duncan, B. B.; Foppa, M.	2013	Epicardial fat: Definition, measurements and systematic review of main outcomes	Literature or systematic review
Bertolami, A.; Lima, J. C.; Cintra, R. M. R.; De Araujo, D. B.; Zats, H. P.; Goncalves, R. M.; Najamura, Y.; Sulzbach, M. L.; Faludi, A.; Bertolami, M. C.; Sposito, A. C.	2015	Adipose tissue dysfunction is associated with increased atherosclerotic burden in individuals with or without weight excess	PRAT not specifically measured
Bertoli, S.; Leone, A.; Krakauer, N. Y.; Bedogni, G.; Vanzulli, A.; Redaelli, V. I.; De Amicis, R.; Vignati, L.; Krakauer, J. C.; Battezzati, A.	2017	Association of Body Shape Index (ABSI) with cardio-metabolic risk factors: A cross-sectional study of 6081 Caucasian adults	PRAT not specifically measured
Bertoli, S.; Leone, A.; Vignati, L.; Bedogni, G.; Martinez-Gonzalez, M. A.; Bes-Rastrollo, M.; Spadafranca, A.; Vanzulli, A.; Battezzati, A.	2015	Adherence to the Mediterranean diet is inversely associated with visceral abdominal tissue in Caucasian subjects	PRAT not specifically measured
Bertoli, S.; Leone, A.; Vignati, L.; Spadafranca, A.; Bedogni, G.; Vanzulli, A.; Rodeschini, E.; Battezzati, A.	2016	Metabolic correlates of subcutaneous and visceral abdominal fat measured by ultrasonography: a comparison with waist circumference	PRAT not specifically measured
Bi, X.; Loo, Y. T.; Henry, C. J.	2018	Ultrasound measurement of intraabdominal fat thickness as a predictor of insulin resistance and low HDL cholesterol in Asians	PRAT not specifically measured
Bignotto, M.; Dei Cas, M.; Paroni, R.; Bianco, E.; Zermiani, P.; Gangale, M. G.; Zadro, V.; Maregatti, M.; Piagnani, A.; Russo, A.; Baldassarre, D.; Folli, F.; Battezzati, P. M.; Zuin, M.	2021	CA.ME.LI.A. An epidemiological study on the prevalence of CArdiovascular, MEtabolic, LIver and Autoimmune diseases in Northern Italy	PRAT not specifically measured

Bisschop, C. N. S.; Peeters, P. H. M.; Monninkhof, E. M.; van der Schouw, Y. T.; May, A. M.	2013	Associations of visceral fat, physical activity and muscle strength with the metabolic syndrome	PRAT not specifically measured
Bogaert, J.; Francone, M.	2013	Pericardial Disease: Value of CT and MR Imaging	PRAT not specifically measured
Borges, L. S. R.; Resende, E. S.; Veloso, F. C.; Diniz, A. L. D.; Penha-Silva, N.; Casella Filho, A.; Dourado, P. M. M.; Chagas, A. C. P.	2015	Perirenal fat and association with metabolic risk factors: The uberlandia heart study	Conference paper
Bouchi, R.; Takeuchi, T.; Akihisa, M.; Ohara, N.; Nakano, Y.; Nishitani, R.; Murakami, M.; Fukuda, T.; Fujita, M.; Minami, I.; Izumiyama, H.; Hashimoto, K.; Yoshimoto, T.; Ogawa, Y.	2015	High visceral fat with low subcutaneous fat accumulation as a determinant of atherosclerosis in patients with type 2 diabetes	Erroneous study design
Brenes-Martín, F.; Melero-Jiménez, V.; López-Guerrero, M. A.; Calero-Ruiz, M. M.; Vázquez-Fonseca, L.; Abalos-Martínez, J.; Quintero-Prado, R.; Torrejón, R.; Visiedo, F.; Bugatto, F.	2023	First Trimester Evaluation of Maternal Visceral Fat and Its Relationship with Adverse Pregnancy Outcomes	PRAT not specifically measured
Brouwer, B. G.; Visseren, F. L. J.; Stolk, R. P.; van der Graaf, Y.;	2007	Abdominal fat and risk of coronary heart disease in patients with peripheral arterial disease	PRAT not specifically measured
Bubnov, R.	2023	Ultrasound Imaging Patterns of Kidney Disease in Patients with Metabolic Syndrome	Conference paper
Bubnov, R.; Kalika, L.; Spivak, M.	2019	Ultrasound evaluation of visceral fat and posture: relevance for diagnosis metabolic syndrome and obesity	Conference paper
Bubnov, R.; Spivak, M.	2019	Ultrasound evaluation of visceral fat and posture for stratification patients with metabolic syndrome	Conference paper
Buyukkaya, R.; Besir, F. H.; Yazgan, S.; Karatas, A.; Kose, S. A.; Aydin, Y.; Erdogmus, B.	2014	The evaluation of carotid intima-media thickness and visceral obesity as an atherosclerosis predictor in newly-diagnosed polycystic ovary syndrome	PRAT not specifically measured
Capelo, A. V.; da Fonseca, V. M.; Peixoto, M. V.; de Carvalho, S. R.; Azevedo, C. M.; Elsas, M. I.; Marques, B.	2016	Visceral adiposity is associated with cytokines and decrease in lung function in women with persistent asthma	PRAT not specifically measured
Capelo, A. V.; Da Fonseca, V. M.; Peixoto, M. V.; De Carvalho, S. R.; Azevedo, C. M.; Elsas, M. I.; Marques, B.	2019	Visceral adiposity is associated with cytokines and decrease in lung function in women with persistent asthma	Conference paper
Cetin, N.; Tatar, I. G.; Yuceege, M.; Ergun, O.; Hekimoglu, B.	2019	Ultrasonographic evaluation of abdominal wall fat index, carotid intima-media thickness and plaque score in obstructive sleep apnea syndrome	PRAT not specifically measured

Chatzistamatiou, E.; Moustakas, G.; Memo, G.; Konstantinidis, D.; Mpampatzeva Vagena, I.; Manakos, K.; Traxanas, K.; Vergi, N.; Feretou, A.; Kallikazaros, I.	2014	Ultrasonography in abdominal adiposity as determinants of cardiovascular risk	Conference paper
Cho, D. H.; Kim, M. N.; Joo, H. J.; Shim, W. J.; Lim, D. S.; Park, S. M.	2019	Visceral obesity, but not central obesity, is associated with cardiac remodeling in subjects with suspected metabolic syndrome	Erroneous study design
Clerte, M.; Baron, D. M.; Flynn, A.; Raher, M. J.; Bloch, K. D.; Picard, M. H.; Buys, E. S.; Brouckaert, P.; Scherrer-Crosbie, M.	2011	Detection of brown adipose tissue activation and mass using contrast ultrasound	Conference paper
Cominacini, M.; Fumaneri, A.; Ballerini, L.; Braggio, M.; Valenti, M. T.; Carbonare, L. D.	2023	Unraveling the Connection: Visceral Adipose Tissue and Vitamin D Levels in Obesity	Erroneous study design
Costa, K. C. M.; Del Ciampo, L. A.; Silva, P. S.; Lima, J. C.; De Paula Martins, W.; Nogueira-De-Almeida, C. A.	2018	Ultrasonographic markers of cardiovascular disease risk in obese children	Paediatric population
Crnobrnja, V.; Ilincic, B.; Stokic, E.; Basta-Nikolic, M.; Slankamenac, S.; Milosavljevic, A. S.; Zeravica, R.; Cabarkapa, V.	2020	Association between ultrasonographically measured visceral fat tissue thickness and proinflammatory adipokines in obesity	PRAT not specifically measured
da Silva, N. F.; Pinho, C. P. S.; da Silva Diniz, A.	2023	Evaluation of ultrasonographic approaches aimed at determining distinct abdominal adipose tissue depots	PRAT not specifically measured
De Mutsert, R.; Den Heijer, M.; Rabelink, T. J.; Smit, J. W. A.; Romijn, J. A.; Jukema, J. W.; De Roos, A.; Cobbaert, C. M.; Kloppenburg, M.; Le Cessie, S.; Middeldorp, S.; Rosendaal, F. R.	2013	The Netherlands epidemiology of obesity (NEO) study: Study design and data collection	PRAT not specifically measured
Dehghan, P.; Eshaghzadeh, M.; Davand, S.; Kouchaki, S.; Langroudi, T.	2023	Relationship Between Common Carotid Intima-Media Thickness and Abdominal Visceral Adipose Tissue, Abdominal Subcutaneous Adipose Tissue, and Neck Subcutaneous Adipose Tissue as Measured by Ultrasonography	PRAT not specifically measured
Delgado-Frias, E.; Gonzalez-Gay, M. A.; Muniz-Montes, J. R.; Gomez Rodriguez-Bethencourt, M. A.; Gonzalez-Diaz, A.; Diaz-Gonzalez, F.; Ferraz-Amaro, I.	2015	Relationship of abdominal adiposity and body composition with endothelial dysfunction in patients with rheumatoid arthritis	Erroneous study design
Demirbas, B.; Gursoy, G.; Simsek, M.; Bahsi, R.; Kosar, P.; Usta, B. M.	2016	Visceral obesity, may have different effects on metabolic syndrome parameters in women and men	PRAT not specifically measured
Depergola, G.; Triggiani, V.; Giorgino, F.; Cospite, M. R.; Garruti, G.; Cignarelli, M.; Guastamacchia, E.; Giorgino, R.	1994	The free testosterone to dehydroepiandosterone-sulfate molar ratio as a marker of visceral fat accumulation in perimenopausal obese women	Study outside criteria dates
Druzhilov, M. A.; Kuznetsova, T. Y.	2019	Internal obesity as a risk factor for arterial hypertension	Conference paper

Elmacl, A. M.; Alp, H.; Donmez, M. I.	2022	Evaluation of subclinical cardiovascular risk and cardiac function in children with vesicoureteral reflux: a prospective study	Unsuitable patient population
Fang, H.; Berg, E.; Cheng, X.; Shen, W.	2018	How to best assess abdominal obesity	Literature or systematic review
Ferreira, J.	2018	Role of adipose tissue and skeletal muscle in macrovascular atherosclerotic occlusive disease-peripheral arterial disease and carotid artery disease	Erroneous study design
Fromm, A.; Thomassen, L.; Naess, H.; Waje-Andreassen, U.	2012	Generalized atherosclerotic disease among young ischemic stroke patients. Assessment by the NOR-SYS research protocol. The NORwegian Stroke in the Young study	PRAT not specifically measured
Georgiopoulos, G.; Kontogiannis, C.; Stakos, D.; Bakogiannis, C.; Koliviras, A.; Kyrkou, A.; Karapanou, L.; Benekos, K.; Augoulea, A.; Armeni, E.; Laina, A.; Stellos, K.; Lambrinoudaki, I.; Stamatelopoulos, K.	2018	Abdominal Fat Tissue Echogenicity: A Marker of Morbid Obesity	PRAT not specifically measured
Ghosh, S.; Dey Hazra, O.	2020	Visceral adipose tissue predicts atherosclerotic cardiovascular disease in overweight and obese patients with greater impact on younger individuals	Conference paper
Giannini, L.; Ballerini, L.; Milia, A.; Berni, A.; Poggesi, L.; Boddi, M.	2014	Cardio-renal ectopic fat and organ damage in healthy subjects	Conference paper
Golia, E.; Limongelli, G.; Natale, F.; Fimiani, F.; Maddaloni, V.; Russo, P. E.; Riegler, L.; Bianchi, R.; Crisci, M.; Di Palma, G. D.; Golino, P.; Russo, M. G.; Calabro, R.; Calabro, P.	2014	Adipose tissue and vascular inflammation in coronary artery disease	Erroneous study design
Grunnet, L. G.; Bygbjerg, I. C.; Mutabingwa, T. K.; Lajeunesse-Trempe, F.; Nielsen, J.; Schmiegelow, C.; Vaag, A. A.; Ramaiya, K.; Christensen, D. L.	2022	nfluence of placental and peripheral malaria exposure in fetal life on cardiometabolic traits in adult offspring	PRAT not specifically measured
Guclu, A.; Dursun, B.; Rota, S.; Sabir, N.; Kaya, C.; Yaman, F.	2016	The relationship between visceral adipose tissue and intima-media thickness in patients with kidney disease	PRAT not specifically measured
Guclu, A.; Dursun, B.; Rota, S.; Sabir, N.; Kaya, C.; Yaman, F.	2016	The relationship between visceral adipose tissue and intima-media thickness in patients with kidney disease	PRAT not specifically measured
Haberka, M.; Banska, K.; Gasior, Z.	2015	Ultrasound indexes of adipose tissue and lipid goals attainment in high and very high cardiovascular risk patients	Conference paper

Haberka, M.; Biedron, M.; Gasior, Z	2017	The new cardiometabolic vascular index and coronary artery disease	Conference paper
Haberka, M.; Gasior, Z.	2015	Carotid extra-media thickness in obesity and metabolic syndrome: Anovel index of perivascular adipose tissue. Extra-media thickness in obesity and metabolic syndrome	PRAT not specifically measured
Haberka, M.; Gasior, Z.	2015	A carotid extra-media thickness, PATIMA combined index and coronary artery disease: Comparison with well-established indexes of carotid artery and fat depots	PRAT not specifically measured
Haberka, M.; Matla, M.; Siniarski, A.; Stepien, K.; Malinowski, K. P.; Kubicius, A.; Gasior, Z	2022	Cardiometabolic predictive value of anthropometric parameters, vascular ultrasound indexes, and fat depots in patients at high cardiovascular risk: an 8-year prospective cohort study	PRAT not specifically measured
Haberka, M.; Okopien, B.; Gasior, Z	2016	Obesity, ultrasound indexes of fat depots and lipid goal attainment in patients with high and very high cardiovascular risk: A novel approach towards better risk reduction	PRAT not specifically measured
Haberka, M.; Okopien, B.; Gasior, Z.	2016	Obesity, ultrasound indexes of fat depots and lipid goal attainment in patients with high and very high cardiovascular risk: A novel approach towards better risk reduction	PRAT not specifically measured
Haberka, M.; Skilton, M.; Biedron, M.; Szostak-Janiak, K.; Partyka, M.; Matla, M.; Gasior, Z	2019	Obesity, visceral adiposity and carotid atherosclerosis	PRAT not specifically measured
Haberka, M.; Stanisz-Kempa, J.; Gasior, Z.	2015	Ultrasound fat indexes and coronary artery disease: Improved risk stratification in obese patients with high and very high cardiovascular risk	Conference paper
Haberka, M.; Stolarz-Skrzypek, K.; Biedron, M.; Szostak-Janiak, K.; Partyka, M.; Olszanecka-Glinianowicz, M.; Gasior, Z.	2018	Obesity, visceral fat, and hypertension-related complications	PRAT not specifically measured
Haehn, D. A.; Bajalia, E. M.; Cockerill, K. J.; Kahn, A. E.; Ball, C. T.; Thiel, D. D.	2021	Validation of the Mayo Adhesive Probability score as a predictor of adherent perinephric fat and outcomes in open partial nephrectomy	Erroneous study design
Haghighi-Morad, M.; Shakoori, A.; Salevatipour, B.	2019	Evaluation of abdominal obesity using ultrasound and its correlation with intima media thickness in carotid arteries	PRAT not specifically measured

Hamagawa, K.; Matsumura, Y.; Kubo, T.; Hayato, K.; Okawa, M.; Tanioka, K.; Yamasaki, N.; Kitaoka, H.; Yabe, T.; Nishinaga, M.; Doi, Y. L.	2010	Abdominal visceral fat thickness measured by ultrasonography predicts the presence and severity of coronary artery disease	PRAT not specifically measured
Hannukainen, J. C.; Guzzardi, M. A.; Virtanen, K. A.;	2014	Imaging of Organ Metabolism in Obesity and Diabetes: Treatment Perspectives	PRAT not specifically measured
Hassan, N. E.; El-Masry, S. A.; El-Saeed, G. S. M.; El Hussieny, M. S.	2021	Lipid accumulation product as an index for visceral obesity and cardiovascular risk among a sample of obese egyptian women	PRAT not specifically measured
Hiremath, R.; Ibrahim, J.; Prasanthi, K.; Reddy, H. T.; Shah, R. S.; Haritha, C.	2017	Comparative Study of Ultrasonographic and Anthropometric Measurements of Regional Adiposity in Metabolic Syndrome	PRAT not specifically measured
Hong, S. J.; Park, J. H.; Lim, D. S	2015	Correlation between visceral fat and coronary artery calcium in patients with metabolic syndrome	Conference paper
Iacobellis, G	2005	Imaging of visceral adipose tissue: an emerging diagnostic tool and therapeutic target	Study outside criteria dates
Imahori, Y.; Mathiesen, E. B.; Morgan, K. E.; Frost, C.; Hughes, A. D.; Hopstock, L. A.; Johnsen, S. H.; Emaus, N.; Leon, D. A.	2020	The association between anthropometric measures of adiposity and the progression of carotid atherosclerosis	PRAT not specifically measured
Jaspers, N. E. M.; Van Der Graaf, Y.; Visseren, F. L. J.	2016	Influence of body-mass index and fat distribution on risk of mortality and cardiovascular events in patients with cerebrovascular disease	Conference paper
Kadhem, S.; Bejjanki, H.; Koratala, A.	2019	The "double-line" sign to identify perirenal fat pad: A must-know sonographic sign	PRAT not specifically measured
Kalapur, S.; Singh, C.	2022	Correlation of Visceral Adiposity Index with Visceral Fat in Obese Patients with and without Type 2 Diabetes Mellitus	Conference paper
Kanhai, D. A.	2011	The risk of general and abdominal adiposity in the occurrence of new vascular events and mortality in patients with various manifestations of vascular disease	Conference paper
Kardassis, D.; Schonander, M.; Sjostrom, L.; Karason, K.	2014	Carotid artery remodelling in relation to body fat distribution, inflammation and sustained weight loss in obesity	PRAT not specifically measured
Kataoka, Y.; Nicholls, S. J.	2014	Imaging of atherosclerotic plaques in obesity: excessive fat accumulation, plaque progression and vulnerability	Literature or systematic review
Katsiki, Niki; Dimitriadis, George; Mikhailidis, Dimitri P.	2019	Perirenal Adiposity and Other Excessive Intra- and Peri-Organ Fat Depots: What Is the Connection?	Editorial

Kawada, T.	2016	Letter to the Editor: Ultrasound assessment of abdominal visceral adipose tissue for cardiovascular risk	Editorial
Kawasaki, S.; Aoki, K.; Hasegawa, O.; Numata, K.; Tanaka, K.; Shibata, N.; Shimada, S.; Okamura, A.; Terauchi, Y.	2008	Sonographic evaluation of visceral fat by measuring para- and perirenal fat	Study outside criteria dates
Kennedy, S.; Shepherd, J.; Heymsfield, S.	2020	Prediction of cardiovascular disease risk from clinically available measures brooke smith	Conference paper
Kim, S. K.; Hur, K. Y.; Ahn, C. W.; Cha, B. S.; Nam, M. S.; Lim, S. K.; Kim, K. R.; Lee, H. C.; Huh, K. B.	2003	Intra-abdominal fat distance (IAFD) measured by ultrasonography can estimate not only visceral adiposity, but also cardiovascular risk in patients with type 2 diabetes	Study outside criteria dates
Kim, S. R.; Lerman, L. O	2018	Diagnostic imaging in the management of patients with metabolic syndrome	Literature or systematic review
Kishida, K.; Funahashi, T.; Shimomura, I.	2012	Clinical importance of assessment of type 2 diabetes mellitus with visceral obesity. A Japanese perspective	PRAT not specifically measured
Kunesova, M.; Hainer, V.; Hergetova, H.; Zak, A.; Parizkova, J.; Horejs, J.; Stich, V.	1995	Simple anthropometric measurements--relation to body fat mass, visceral adipose tissue and risk factors of atherogenesis	Study outside criteria dates
Lam, J. K. Y.; Lam, K. S. L.; Chow, W. S.; Tan, K. C. B	2014	A middle-aged man with increasing body fat	PRAT not specifically measured
Lear, S. A.; Sarna, L. K.; Siow, T. J.; Mancini, G. B.; Siow, Y. L.; O, K.	2012	Oxidative stress is associated with visceral adipose tissue and subclinical atherosclerosis in a healthy multi-ethnic population	PRAT not specifically measured
Lee, M. J.; Shin, D. H.; Kim, S. J.; Oh, H. J.; Yoo, D. E.; Kim, J. K.; Park, J. T.; Han, S. H.; Kang, S. W.; Choi, K. H.; Yoo, T. H.	2012	Visceral fat thickness is associated with carotid atherosclerosis in peritoneal dialysis patients	PRAT not specifically measured
Leonardo, R.; Resende, E. S.; Penha-Silva, N.; Roerver-Borges, A. S.; Casella-Filho, A.; Diniz, A. L. D.; Dourado, P. M. M.; Denardi, C.; Veloso, F.; Silva, M.; Chagas, A. C. P.	2014	Ectopic visceral fat and differences between risk factors and coronary syndromes	Conference paper
Lim, S.; Meigs, J. B.	2014	Links between ectopic fat and vascular disease in humans	Literature or systematic review
Lim, S.; Meigs, J. B.	2013	Ectopic fat and cardiometabolic and vascular risk	Literature or systematic review
Lima, M. M. O.; Pareja, J. C.; Alegre, S. M.; Geloneze, S. R.; Kahn, S. E.; Astiarraga, B. D.; Chaim, E. A.; Baracat, J.; Geloneze, B.	2013	Visceral fat resection in humans: Effect on insulin sensitivity, beta-cell function, adipokines, and inflammatory markers	PRAT not specifically measured

Liu, B. X.; Sun, W.; Kong, X. Q. 2019	2019	Perirenal Fat: A Unique Fat Pad and Potential Target for Cardiovascular Disease	Literature or systematic review
Liu, K. H.; Chan, Y. L.; Chan, W. B.; Kong, W. L.; Kong, M. O.; Chan, J. C.	2003	Sonographic measurement of mesenteric fat thickness is a good correlate with cardiovascular risk factors: comparison with subcutaneous and preperitoneal fat thickness, magnetic resonance imaging and anthropometric indexes	Study outside criteria dates
Liu, K. H.; Chan, Y. L.; Chan, W. B.; Kong, W. L.; Kong, M. O.; Chan, J. C. N.	2003	Sonographic measurement of mesenteric fat thickness is a good correlate with cardiovascular risk factors: Comparison with subcutaneous and preperitoneal fat thickness, magnetic resonance imaging and anthropometric indexes	Study outside criteria dates
Liu, K. H.; Chu, W. C. W.; To, K. W.; Ko, F. W. S.; Ng, S. S. S.; Ngai, J. C. L.; Chan, K. P.; Yip, W. H.; Ahuja, A. T.; Hui, D. S. C.	2016	Mesenteric fat thickness is associated with metabolic syndrome independently of Apnoea-Hypopnoea Index in subjects with obstructive sleep apnoea	PRAT not specifically measured
Lo, J.; Dolan, S. E.; Kanter, J. R.; Hemphill, L. C.; Connelly, J. M.; Lees, R. S.; Grinspoon, S. K.	2006	Effects of obesity, body composition, and adiponectin on carotid intima-media thickness in healthy women	Study outside criteria dates
Lu, Q.; Cheng, L.; Wang, T.; Wan, J.; Liao, L.; Zeng, J.; Qin, C.; Li, K	2008	Visceral fat, arterial stiffness, and endothelial function in peritoneal dialysis patients	Study outside criteria dates
Lupattelli, G.; Pirro, M.; Mannarino, M. R.; Siepi, D.; Roscini, A. R.; Schillaci, G.; Mannarino, E.	2012	Visceral fat positively correlates with cholesterol synthesis in dyslipidaemic patients	PRAT not specifically measured
Ma, R. C.; Liu, K. H.; Lam, P. M.; Cheung, L. P.; Tam, W. H.; Ko, G. T.; Chan, M. H.; Ho, C. S.; Lam, C. W.; Chu, W. C.; Tong, P. C.; So, W. Y.; Chan, J. C.; Chow, C. C	2011	Sonographic measurement of mesenteric fat predicts presence of fatty liver among subjects with polycystic ovary syndrome	PRAT not specifically measured
Maimaituxun, Gulinu; Fukuda, Daiju; Izaki, Hirofumi; Hirata, Yoichiro; Kanayama, Hiro-Omi; Masuzaki, Hiroaki; Sata, Masataka; Shimabukuro, Michio	2020	Levels of Adiponectin Expression in Peri-Renal and Subcutaneous Adipose Tissue and Its Determinants in Human Biopsied Samples	Erroneous study design
Mazzoccoli, G.; Dagostino, M. P.; Greco, A.	2011	Age-related changes of epicardial fat thickness	PRAT not specifically measured
McCarthy, E. A.; Strauss, B. J. G.; Walker, S. P.; Permezel, M	2004	Determination of maternal body composition in pregnancy and its relevance to perinatal outcomes	Study outside criteria dates
Milia, A.; Giannini, L.; Ballerini, L.; Berni, A.; Fiorillo, C.; Becatti, M.; Poggesi, L.; Boddi, M	2014	Ultrasonographic abdominal fat assessment in cardiometabolic risk evaluation of healthy subjects	PRAT not specifically measured

Morotti, E.; Giovanni Artini, P.; Persico, N.; Battaglia, C.	2019	Metformin metabolic and vascular effects in overweight/moderately obese hyper insulinemic PCOS patients treated with contraceptive vaginal ring: a pilot study	PRAT not specifically measured
Moss, A.; Sievert, K.; Siegfried, W.; Siegfried, A.; Brandt, S.; Koenig, W.; Wabitsch, M.	2016	Sonographically Assessed Intra-Abdominal Fat and Cardiometabolic Risk Factors in Adolescents with Extreme Obesity	Paediatric population
Moustakas, G.; Chatzistamatiou, E.; Memo, G.; Konstantinidis, D.; Mitatou, Z.; Manakos, K.; Feretou, A.; Syros, P.; Kasakogias, A.; Kallikazaros, I.	2014	General and regional adiposity as determinants of cardiovascular risk	PRAT not specifically measured
Naboush, A.; Hamdy, O.	2013	Measuring visceral and hepatic fat in clinical practice and clinical research	PRAT not specifically measured
Nalini, M.; Sharafkhah, M.; Poustchi, H.; Sepanlou, S. G.; Pourshams, A.; Radmard, A. R.; Khoshnia, M.; Gharavi, A.; Dawsey, S. M.; Abnet, C. C.; Boffetta, P.; Brennan, P.; Sotoudeh, M.; Nikmanesh, A.; Merat, S.; Etemadi, A.; Shakeri, R.; Malekzadeh, R.; Kamangar, F.	2019	Comparing anthropometric indicators of visceral and general adiposity as determinants of overall and cardiovascular mortality	PRAT not specifically measured
Napoli, L.; Tovoli, F.; Ferri, S.; Piscaglia, F.; Bolondi, L.	2017	Ultrasound-measured visceral adipose tissue correlates with risk factors, severity and co-morbidities of NAFLD better than waist circumference	Conference paper
Navarro, E.; Mijac, V.; Ryder, H. F.	2010	Ultrasonography measurement of intrabdominal visceral fat in obese men. Association with alterations in serum lipids and insulinemia	Language other then English
Nazzaro, P.; Caradionna Moscatelli, F.; Nardecchia, A.; Contini, M.; Schirosi, G.; De Benedittis, L.; Papagni, A.	2019	The ultrasound-determined visceral fat is associated to preclinical macro-and micro-vascular damage in overweight hypertensives	Conference paper
Nazzaro, P.; Caradonna Moscatelli, F.; Nardecchia, A.; Contini, M.; Schirosi, G.; De Benedittis, L.; Papagni, A. M.	2018	Visceral fat quantification by echography in overweight hypertensives and cardiovascular risk assessed by arterial tonometry and neuropsychological assessment	Conference paper
Nazzaro, P.; Caradonna Moscatelli, F.; Nardecchia, A.; Contini, M.; Schirosi, G.; De Benedittis, L.; Papagni, A. M.; Vitali, I.; Laselva, G.	2018	The role of ultrasound to quantify the visceral fat in overweight hypertensives and its relationship with the cardiovascular risk	Conference paper
Noites, A.; Moreira, A.; Melo, C.; Faria, M.; Vilarinho, R.; Freitas, C.; Monteiro, P. R. R.; Carvalho, P.; Adubeiro, N.; Amorim, M.; Nogueira, L.; Santos, R	2017	Acute effects of physical exercise with microcurrent in the adipose tissue of the abdominal region: A randomized controlled trial	PRAT not specifically measured

Pradhan, J.; Mishra, I.; Rattan, R.; Choudhury, A. K.; Baliarsinha, A. K.	2022	Correlation of markers of inflammation with hormonal, metabolic parameters, insulin resistance and adiposity indices in first-degree relatives of patient with polycystic ovary syndrome	PRAT not specifically measured
Roccarina, D.; Prat, L. I.; Guerrero, M.; Buzzetti, E.; Mantovani, A.; Goyale, A.; Arico, F.; Saffioti, F.; Pinzani, M.; Tsochatzis, E.	2019	Cardiovascular risk factors and fibrosis severity in NAFLD: is there a link?	Conference paper
Roever, L. S.; Resende, E. S.; Diniz, A. L. D.; Penha-Silva, N.; Veloso, F. C.; Casella, A.; Dourado, P. M. M.; Chagas, A. C. P.	2016	Abdominal Obesity and Association With Atherosclerosis Risk Factors	PRAT not specifically measured
Roever, L.; Resende, E. S.; Diniz, A. L.; Penha-Silva, N.; Veloso, F. C.; Casella-Filho, A.; Dourado, P. M.; Chagas, A. C.	2015	Ectopic adiposopathy and association with cardiovascular disease risk factors: The Uberlandia Heart Study	Editorial
Rossi, A. P.; Harris, T. B.; Fantin, F.; Armellini, F.; Zamboni, M.	2014	The multidomain mobility lab in older persons: From bench to bedside. The assessment of body composition in older persons at risk of mobility limitations	Erroneous study design
Ryabikov, A.; Guliev, Z.; Malyutina, S.; Ragino, Y. U.; Veryovkin, E.	2014	Novel sonographic indicators of body adiposity in relation to cardiometabolic risk factors	Conference paper
Salvetti, G.; Santini, F.; Versari, D.; Virdis, A.; Fierabracci, P.; Scartabelli, G.; Pucci, A.; Galli, G.; Piaggi, P.; Taddei, S.; Vitti, P.; Salvetti, A.; Pinchera, A.	2008	Fat distribution and cardiovascular risk in obese women	Study outside criteria dates
Sandhu, J. S.; Esht, V.; Shenoy, S	2012	Cardiovascular risk factors in middle age obese Indians: a cross-sectional study on association of per cent body fat and intra-abdominal fat mass	PRAT not specifically measured
Sandhu, J. S.; Esht, V.; Shenoy, S.	2014	Cardiovascular risk factors in middle age obese Indians: A cross-sectional study on association of per cent body fat and intra-abdominal fat mass	PRAT not specifically measured
Savchenko, O.; Zavalskaya, T.; Lizogub, V.; Kuzhel, O.; Baitser, M.; Zapeka, Y.	2015	Methods in Abdominal Obesity	Language other then English
Seven, E.; Thuesen, B. H.; Linneberg, A.; Jeppesen, J. L.	2016	Abdominal adiposity distribution quantified by ultrasound imaging and incident hypertension in a general population	PRAT not specifically measured
Shabestari, A. A.; Bahrami-Motlagh, H.; Hosseinpanah, F.; Heidari, K	2013	Abdominal fat sonographic measurement compared to anthropometric indices for predicting the presence of coronary artery disease	PRAT not specifically measured

Shuster, A.; Patlas, M.; Pinthus, J. H.; Mourtzakis, M.	2012	The clinical importance of visceral adiposity: A critical review of methods for visceral adipose tissue analysis	Literature or systematic review
Siervo, M.; Lara, J.; Celis-Morales, C.; Vacca, M.; Oggioni, C.; Battezzati, A.; Leone, A.; Tagliabue, A.; Spadafranca, A.; Bertoli, S.	2016	Age-related changes in basal substrate oxidation and visceral adiposity and their association with metabolic syndrome	PRAT not specifically measured
Smereczynski, A.; Kolaczyk, K.; Bernatowicz, E	2015	Intra-abdominal fat. Part I. The images of the adipose tissue localized beyond organs	PRAT not specifically measured
Smiliansky, N.; Roman, S. S.; Ottati, G.; Bruno, G.; Garau, M.; Noboa, O. A.; Etchegoimberry, V.	2022	Peri and Para-Renal Fat Tissue and Risk of CKD in Morbid Obese Patients	Erroneous study design
Smith-Ryan, A. E.; Blue, M. N. M.; Trexler, E. T.; Hirsch, K. R	2018	Utility of ultrasound for body fat assessment: validity and reliability compared to a multicompartment criterion	PRAT not specifically measured
Stigall, A. N.; Hunter, R. L.; Evans, K. D.; Spees, C. K.	2019	A Longitudinal Study to Assess Abdominal Adiposity by Sonography, DXA, and BMI	PRAT not specifically measured
Tongdee, P.; Loyd, R. A.; Winwan, K.; Nimkuntod, P.	2016	Application of Visceral Adiposity Index and Anthropometry to Identify Preclinical Atherosclerosis in Menopausal Status Women	Conference paper
Tornaghi, G.; Raiteri, R.; Pozzato, C.; Rispoli, A.; Bramani, M.; Cipolat, M.; Craveri, A.	1994	Anthropomorphic or ultrasonic measurements in assessment of visceral fat - a comparative study	Study outside criteria dates
Viscogliosi, Giovanni; Chiriac, Iulia Maria; Andreozzi, Paola; Ettorre, Evaristo	2016	Markers of Visceral Adiposity for Dementia Risk Assessment. The Epicardial Adipose Tissue (EAT) Thickness	Editorial
Vlachos, I. S.; Hatziioannou, A.; Perelas, A.; Perrea, D. N.	2007	Sonographic assessment of regional adiposity	Study outside criteria dates
Von Schnurbein, J.	2011	Sonographically assessed intra-abdominal fat	Editorial
Vos, A. G.	2020	Obesity and the heart: The impact of obesity beyond the body mass index	Editorial
Wang, H.; Chen, Y. E.; Eitzman, D. T.	2014	Imaging body fat: techniques and cardiometabolic implications	Literature or systematic review
Woldemariam, M. M.; Evans, K. D.; Butwin, A. N.; Pargeon, R. L.; Volz, K. R.; Spees, C.	2018	Measuring Abdominal Visceral Fat Thickness With Sonography: A Methodologic Approach	Conference paper
Woldemariam, M. M.; Evans, K. D.; Butwin, A. N.; Pargeon, R. L.; Volz, K. R.; Spees, C.	2018	Measuring Abdominal Visceral Fat Thickness With Sonography: A Methodologic Approach	PRAT not specifically measured
Wykretowicz, M.; Katulska, K.; Krauze, T.; Milewska, A.; Przymuszala, D.; Piskorski, J.; Stajgis, M.; Wysocki, H.	2013	Renal morphology assessed by ultrasound in relation to central haemodynamics and body fat	PRAT not specifically measured

Xu, C.; Zhao, S.; Yu, S.; Chi, C.; Fan, X.; Ji, H.; Maimaitiaili, R.; Teliewubai, J.; Li, X.; Zhang, Y.; Xu, Y.	2021	Association between organ damage and visceral adiposity index in community-dwelling elderly Chinese population: the Northern Shanghai Study	PRAT not specifically measured
Yamashiro, K.; Tanaka, R.; Tanaka, Y.; Miyamoto, N.; Shimada, Y.; Ueno, Y.; Urabe, T.; Hattori, N.	2014	Visceral fat accumulation is associated with cerebral small vessel disease	PRAT not specifically measured
Yu, Q.; Huang, S.; Xu, T. T.; Wang, Y. C.; Ju, S	2021	Measuring Brown Fat Using MRI and Implications in the Metabolic Syndrome	Erroneous study design
Yu, Y.; Zhang, F. L.; Yan, X. L.; Zhang, P.; Guo, Z. N.; Yang, Y.	2021	Visceral adiposity index and cervical arterial atherosclerosis in northeast China: a population based cross-sectional survey	PRAT not specifically measured
Zhou, Y.; Koizumi, N.; Kubota, N.; Asano, T.; Yuhashi, K.; Mochizuki, T.; Kadowaki, T.; Sakuma, I.; Liao, H.	2010	Fast and accurate ultrasonography for visceral fat measurement	Conference paper
